# Molecular interplay between linc01134 and YY1 dictates hepatocellular carcinoma progression

**DOI:** 10.1186/s13046-020-01551-9

**Published:** 2020-04-09

**Authors:** Zhonghou Rong, Zhiyi Wang, Xinxing Wang, Chengkun Qin, Wenmao Geng

**Affiliations:** Department of Hepatobiliary Surgery, Shandong Provincial Hospital Affiliated to Shandong University, Jinan, Shandong 250021 People’s Republic of China

**Keywords:** YY1, linc01134, IGF2BP1, Hepatocellular carcinoma

## Abstract

**Background:**

Revealing the mechanical role of long non-coding RNAs (lncRNAs) in tumorigenesis can contribute to novel therapeutic target for cancers. The regulatory role of linc01134 in hepatocellular carcinoma (HCC) has not been studied yet.

**Materials and methods:**

qRT-PCR and western blot were conducted to measure relevant RNA and protein expressions. CCK-8, colony formation, EdU, flow cytometry, wound-healing, transwell assays and xenograft experiments were performed to determine the role of linc01134 in HCC. ChIP and luciferase reporter assays were performed to analyze the effects of Yin Yang-1 (YY1) on linc01134 transcription activity. Relevant mechanical experiments were performed to verify interaction between relative genes.

**Results:**

YY1 enhanced linc01134 transcription by interacting with linc01134 promoter. Knockdown of linc01134 inhibited proliferation, migration and epithelial-mesenchymal transition (EMT), yet promoting apoptosis in HCC cells. Mechanically, linc01134 acted as miR-324-5p sponge and interacted with insulin-like growth factor 2 mRNA binding protein 1 (IGF2BP1) to increase the stability of YY1 mRNA expression. Up-regulated YY1 continuously stimulated linc01134 expression by enhancing linc01134 promoter activity, forming a positive feedback loop.

**Conclusion:**

Linc01134/miR-324-5p/IGF2BP1/YY1 feedback loop mediates HCC progression, which possibly provide prognosis and treatment target of HCC.

## Background

With approximately 850, 000 new diagnosis cases each year, liver cancer is the second leading cause of cancer-related death globally [1]. Hepatocellular carcinoma (HCC) is the most prevalent subtype of liver cancer, accounting for about 90% amongst liver cancer cases. Although advance in therapeutic methods has been made in recent years, the mortality of HCC still ranks the third among all cancers. Worse still, this rate is still on the rise worldwide [2]. Frequent recurrence and metastasis are major reasons for the high mortality of HCC. From molecular level, the unclear pathogenesis behind HCC is partially responsible for the clinical treatment stagnancy. Hence, in-depth study of the molecular mechanisms behind HCC tumorigenesis is of vital significance.

In human genome, the majority of transcripts are non-coding RNAs, while only approximately 1.2% of these transcripts represent protein-coding genes [3]. The numerous non-protein-coding transcripts have been discovered to play important regulatory role in diseases, including cancers. Among these, long non-coding RNAs (lncRNAs), which are over 200 nucleotides in length, have emerged as key regulators in biological processes for their diverse and complex work mechanisms [4]. LncRNAs have been reported to affect an array of cellular functions, such as cellular growth, apoptosis, migration and invasion, as well as EMT progression [5–8]. Also, increasing lncRNAs have been detected to be aberrantly expressed in cancers. Besides, the expression of lncRNAs could be impacted by transcription factors or pioneer factor. Transcription factor or pioneer factor induced lncRNA promoter region activation or silence to further alter the cellular function elicited by lncRNAs. For instance, SP1 transcriptionally activated lncRNA ZFAS1 to accelerate colorectal cancer progression via targeting downstream miR-150-5p/VEGFA axis [9]. Yin Yang-1 (YY1) is a multifunctional transcription factor that can promote or suppress the promoter activity of various genes. Additionally, YY1 has been reported to be involved in the EMT progression and biological functions in colorectal cancer [10]. YY1 was also erratically expressed in HCC and aggravate its progression. For instance, YY1 enrichment contributed to EZH2 recruitment for H3K27me3-regulated microRNAs silence, consequently activating NF-κB signaling in HCC carcinogenesis [11].

Recently, growing number of lncRNAs have been reported to function as miRNAs sponges and further mediate the target of miRNAs, constituting a competing endogenous RNA (ceRNA) network. This post-transcriptional molecular mechanism of lncRNAs has gained increasing attention [12, 13]. Cytoplasmic lncRNA can restore the expression of target gene via sponging miRNA, thus mediating the cellular function of target gene. Of note, lncRNAs are also elucidated to modulate gene expression via interacting with RNA binding proteins (RBPs). For instance, LINC01093 curbed HCC progression by interacting with IGF2BP1 and interfering the binding between IGF2BP1 and GLI1, thus impacting expression of GLI1 downstream molecules associated with HCC progression [14]. Linc01134 is a novel identified long non-coding RNA without the potential to encode proteins. However, its molecular role in cancer has not been studied yet. Whether linc01134 regulates the progression of HCC via mediating the expression of target genes remain unclear. In this study, we investigated underlying mechanism of linc01134 in HCC tumorigenesis and progression.

## Material and methods

### Tissue specimens

This research was conducted under the approval and supervision of the Ethics Committee of Shandong Provincial Hospital Affiliated to Shandong University 46 recruited patients without any preoperative chemotherapy or radiation therapy had signed the written informed consent before study. After surgical resection, both HCC tissues and para-tumor tissues were instantly stored at − 80 °C in liquid nitrogen till RNA extraction.

### Cell culture and treatment

Normal liver cell (QSG-7701) and HCC cells (SNU-449, HepG2, HLF, Huh-7) were purchased from the American Type Culture Collection (ATCC; Manassas, VA, USA) and cultured at 37 °C in a humidified atmosphere containing 5% CO_2_. DMEM (Gibco, Grand Island, NY, USA) adding antibiotics (Gibco) and 10% FBS (Gibco) were utilized for culturing cells. The RNA polymerase II inhibitor, α-amanitin (100 nM, Sigma-Aldrich, St. Louis, MO, USA) was added into culture medium for treating cells.

### Cell transfection

Specific shRNAs against linc01134 (sh-linc01134#1/2), YY1 (sh-YY1) or IGF2BP1 (sh-IGF2BP1) and the non-specific shRNAs as negative control (sh-NC), along with the pcDNA3.1/YY1, pcDNA3.1/linc01134 or pcDNA3.1/IGF2BP1 and the empty vectors as control, were constructed by Genechem (Shanghai, China). MiR-324-5p mimics and NC mimics were from GenePharma (Shanghai, China). SNU-449 or HepG2 cells were transfected individually with these plasmids via Lipofectamine 3000 (Invitrogen, Carlsbad, CA, USA). The transfected cells were collected and the stably transfected cells were selected out after 48 h of 2 μg/ml puromycin treatment.

### Quantitative real-time polymerase chain reaction (qRT-PCR)

SNU-449 or HepG2 cells were re-suspended in 1 mL of TRIzol (Invitrogen), total cellular RNA was isolated, and reverse transcribed into cDNA. Gene expression was detected by qRT-PCR. GAPDH or U6 acted as endogenous control.

### Cell counting Kit-8 (CCK-8) assay

1 × 10^3^ transfected SNU-449 or HepG2 cells were added to 96-well plates before CCK-8 (Beyotime, Jiangsu, China) was added. Absorbance at 450 nm was read via microplate reader (bio-rad, Hercules, CA, USA)

### Colony formation assay

Transfected SNU-449 or HepG2 cells were planted in 24-well plates (500 cells/well). Colonies were fixed for 15 min by 100% methanol (Sigma-Aldrich) following 14-day culturing. Cells were stained for 20 min utilizing 0.5% crystal violet (Sigma-Aldrich). Colonies with > 50 cells were counted manually.

### EdU staining

EdU staining was undertaken with a Click iT™ EdU cell proliferation assay kit (Invitrogen). Cell nuclei were double-dyed for 10 min using DAPI (Sigma-Aldrich) and observed via florescence microscope (Olympus, Tokyo, Japan).

### Flow cytometer

Annexin V-FITC/PI Apoptosis Detection Kit (Sigma-Aldrich) was applied to stain transfected SNU-449 or HepG2 cells in the dark. The apoptotic rate was assayed with flow cytometer (BD Biosciences, Franklin Lakes, NJ, USA) and CellQuest software (BD Biosciences).

### Xenograft tumors

Animal study was undertaken with the approval of the Animal Research Ethics Committee of Shandong Provincial Hospital Affiliated to Shandong University BALB/c nude mice (6 weeks old) were bought from Shi Laike Company (Shanghai, China). SNU-449 cells transfected with sh-linc01134 or sh-NC were injected subcutaneously to animals. Tumors volume was assessed every 4 days. Following 4 weeks, mice were killed. Tumors were harvested and weighed.

### Immunohistochemistry (IHC)

Xenograft tumor tissue samples were immunostained for Ki-67 with the specific antibody from Santa Cruz Biotechnology (Dallas, TX, USA) as per the established protocol.

### Transwell assay

Transfected cells in serum-free medium were planted into the Transwell upper chambers (Corning, Cambridge, MA, USA). 10% FBS-medium was added to the bottom chambers. In invasion assay, upper chambers were pre-coated with Matrigel (BD Biosciences). 48 h later, migratory cells were fixed, stained and counted under microscope (Olympus).

### Wound-healing assay

Transfected SNU-449 or HepG2 cells were cultured in 6-well plates to 90% confluence. Wound was made utilizing pipette tip. The widths of the wounds were examined at 0 and 24 h.

### Immunofluorescence (IF)

Transfected SNU-449 or HepG2 cells were incubated in sequence with the specific primary antibodies to E-cadherin (Abcam) and N-cadherin (Abcam), as well as secondary antibodies. Hoechst 33342 (Thermo Fisher Scientific, Waltham, MA, USA) was used for counterstaining nuclei.

### Western blot

Total cellular protein samples were separated with 10% SDS PAGE and transferred electrophoretically onto PVDF membranes (Millipore, Billerica, MA, USA). Primary antibodies against E-cadherin (ab40772), N-cadherin (ab76057), Vimentin (ab8978, Abcam), IGF2BP1 (ab82968, Abcam), YY1 (ab109228, Abcam), CDK4 (ab199728, Abcam), cyclin D1 (ab16663, Abcam), CDK2 (ab32147, Abcam) and GAPDH (ab9485, Abcam), along with HRP-conjugated secondary antibody were obtained and employed. GAPDH served as internal control.

### Haematoxylin and eosin (HE) staining

Xenograft tumor tissue samples were fixed and embedded by paraffin. Sample sections with 4 μm of thickness were utilized for HE staining (Sigma-Aldrich).

### Chromatin Immunoprecipitation (ChIP)

Chromatin was crosslinked and sonicated to fragments (500 bp). Immunoprecipitation was implemented by magnetic beads coated with anti-YY1 or anti-IgG (Millipore). Precipitated DNA fragments were studied with qRT-PCR following total RNA extraction.

### Luciferase reporter assay

The pGL3-linc01134 promoter vector was constructed via cloning the wild-type or mutant interacting sequences of YY1 in linc01134 promoter into pGL3-Basis vector (Promega, Madison, WI, USA), co-transfected into with sh-NC and sh-YY1 or pcDNA 3.1 and pcDNA3.1/YY1. The wild-type or mutant sequences of miR-324-5p in linc01134 or IGF2BP1 were sub-cloned into pmirGLO dual-luciferase vector (Promega), co-transfected with miR-324-5p mimics or NC mimics into HEK-293 T, SNU-449 or HepG2 cells. Fourty-eight hour later, dual luciferase reporter assay system (Promega) was applied for examination of luciferase activities.

### Subcellular fractionation

Cytoplasmic and Nuclear RNA Purification Kit was acquired commercially from Norgen (Thorold, ON, Canada). Expressions of GAPDH, U6 and linc01134 in nuclear and cytoplasm fractions of SNU-449 or HepG2 cells were examined via qRT-PCR.

### Fluorescence in situ hybridization (FISH)

Cy3-labeled Linc01134 probe was synthesized by RiboBio. Fluorescent in Situ Hybridization Kit (RiboBio) was used to assess fluorescence signal. Cell nuclei were counterstained using Hoechst.

### RNA pull-down

Cell lysates were individually incubated with biotinylated RNAs, followed by adding magnetic beads. The RNA enrichment was evaluated by qRT-PCR or western blotting.

### RNA Immunoprecipitation (RIP)

RIP was undertaken in SNU-449 or HepG2 cells using Magna RIP™ RNA-Binding Protein Immunoprecipitation Kit (Millipore) and antibody against normal mouse IgG as control. Finally, qRT-PCR analysis determined the RNA enrichment in the immunoprecipitated complex.

### Cell cycle analysis

A single-cell suspension was prepared for SNU449 and HepG2 cells in ice-cold phosphate-buffered saline (PBS). Next, the cells were fixed with 70% ethanol for 12 h at 4 °C. Cell clumps were removed by passing-through a cell strainer (BD BioSciences, CA, USA). Four-hundred microliters of propidium iodide (0.1 mg/mL) were incubated for 10 min and detected by flow cytometry and DNA content data was detected using BD Accuri Cytometry (BD BioSciences, Franklin Lakes, NJ, USA).

### Statistical analysis

Data of at leastthree independent replications were analyzed by SPSS Vision 19.0 (SPSS, Chicago, IL, USA) and showed as means ± SD. Pearson’s correlation coefficient was adopted for evaluating the gene expression correlations. Differences in groups were evaluated by Student’s t-test or ANOVA, with *p*-value less than 0.05 as statistical significance.

## Results

### Knockdown of linc01134 can dampen HCC cell proliferation in vitro and suppress tumor growth in vivo

**Fig. 1:**
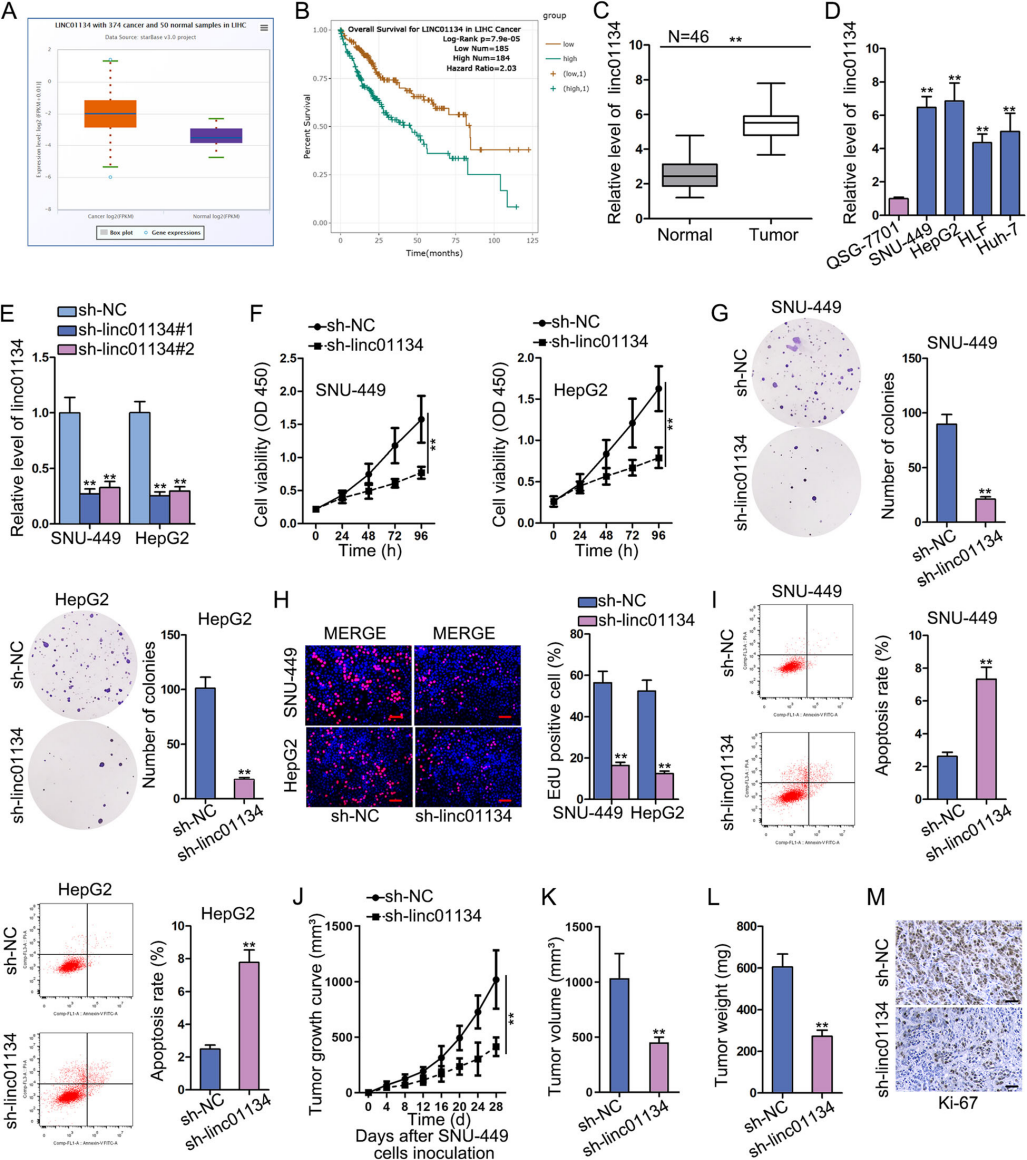
Knockdown of linc01134 can dampen HCC cell proliferation in vitro and suppress tumor growth in vivo. **a** The expression of linc01134 in LIHC tissues and para-tumor tissues based on starBase. **b** Kaplan-Meier analysis demonstrated the effects of linc01134 expression on LIHC patients’ overall survival. **c** qRT-PCR assay detected linc01134 expression in HCC tissues and para-tumor tissues. **d** Linc01134 expression in one normal cell line (QSG-7701) and four HCC cell lines (SNU-449, HepG2, HLF and Huh-7) was analyzed by qRT-PCR. **e** qRT-PCR was used to evaluate the transfection efficiency of linc01134 knockdown. **f** CCK-8 was performed to study cell viability after knockdown of linc01134. **g**-**h**. Colony formation and EdU were performed to assess cell proliferation when down-regulating linc01134. **i** Flow cytometry evaluated cell apoptosis after knockdown of linc01134. **j** Xenograft growth curve between sh-NC and sh-linc01134 groups. **k**-**l** Tumor volume and weight were measured between sh-NC and sh-linc01134 groups. **m** IHC analysis was performed to detect Ki-67 positive cells. **P < 0.01

To begin with, we analyzed the expression status of linc01134 in liver hepatocellular carcinoma (LIHC) tissues and para-tumor tissues samples from starBase. It can be noticed that linc01134 was remarkably overexpressed in LIHC tissues compared with normal tissue (Fig. 1a). Several databases manifested the significantly low expression of linc01134 in normal liver tissues (Fig. S1A, S1B, S1C). Besides, linc01134 was presented by LncBook (https://bigd.big.ac.cn/lncbook/index) to have no protein-coding potential (Fig. S1D). Kaplan-Meier survival analysis found that high expression of linc01134 was closely associated with shorter overall survival, while low expression of linc01134 had the opposite outcome (Fig. 1b). We then analyzed the expression of linc01134 in 46 paired collected HCC tissues and normal tissue. qRT-PCR result manifested that linc01134 was overtly up-regulated in HCC tissues (Fig. 1c). Likewise, qRT-PCR data also demonstrated an aberrant up-regulation of linc01134 in HCC cell lines (SNU-449, HepG2, HLF and Huh-7) compared with normal liver epithelial cell line (QSG-7701) (Fig. 1d). Based on the above data, we assumed that linc01134 might play a role in HCC. To verify this assumption, we knocked down linc01134 in SNU-449 and HepG2 cells to study its influence on cellular behavior. By transfecting sh-linc01134#1/2, we got low expression of linc01134 in SNU-449 and HepG2 (Fig. 1e). According to CCK-8 result, knockdown of linc01134 remarkably reduced cell viability compared with control group (Fig. 1f). Meanwhile, silencing linc01134 could suppress cell proliferation by counting colony cells and calculating EdU positive cell rate (Fig. 1g, h). Besides, PI-FACS analysis showed that linc01134 knockdown increased the percentage of cells at the G0/G1 phase, while decreased that in S and G2/M phases (Fig. S2A). We measured the mRNA and protein levels of CDK4, cyclin D1, CDK2, which were associated with cell cycle. Data presented that all these three genes were down-regulated by linc01134 depletion, suggesting that sh-linc01134 could induce cell cycle arrest (Fig. S2B). We performed flow cytometry to examine the effect of sh-linc01134 on cell apoptosis. An increase in the fraction of apoptotic cells was observed in cells transfected with sh-linc01134 (Fig. 1i). We used xenografts to study the effects of linc01134 on tumor growth in vivo. The tumor growth speed slowed down in sh-linc01134 group in comparison with control group (Fig. 1j). We found that the tumors in sh-linc01134 group were obviously smaller and lighter compared with those in control group (Fig. 1k, l). Consistently, immunohistochemistry (IHC) result revealed that Ki-67 positive cells were conspicuously decreased in sh-linc01134 group than control group (Fig. 1m). In short, linc01134 was up-regulated in HCC tissues and cell lines. Linc01134 down-regulation hampered HCC cell proliferation in vitro and repressed tumor growth in vivo.

### Knockdown of linc01134 can curb HCC cell migration, invasion and EMT process in vitro and impede metastasis in vivo

**Fig. 2:**
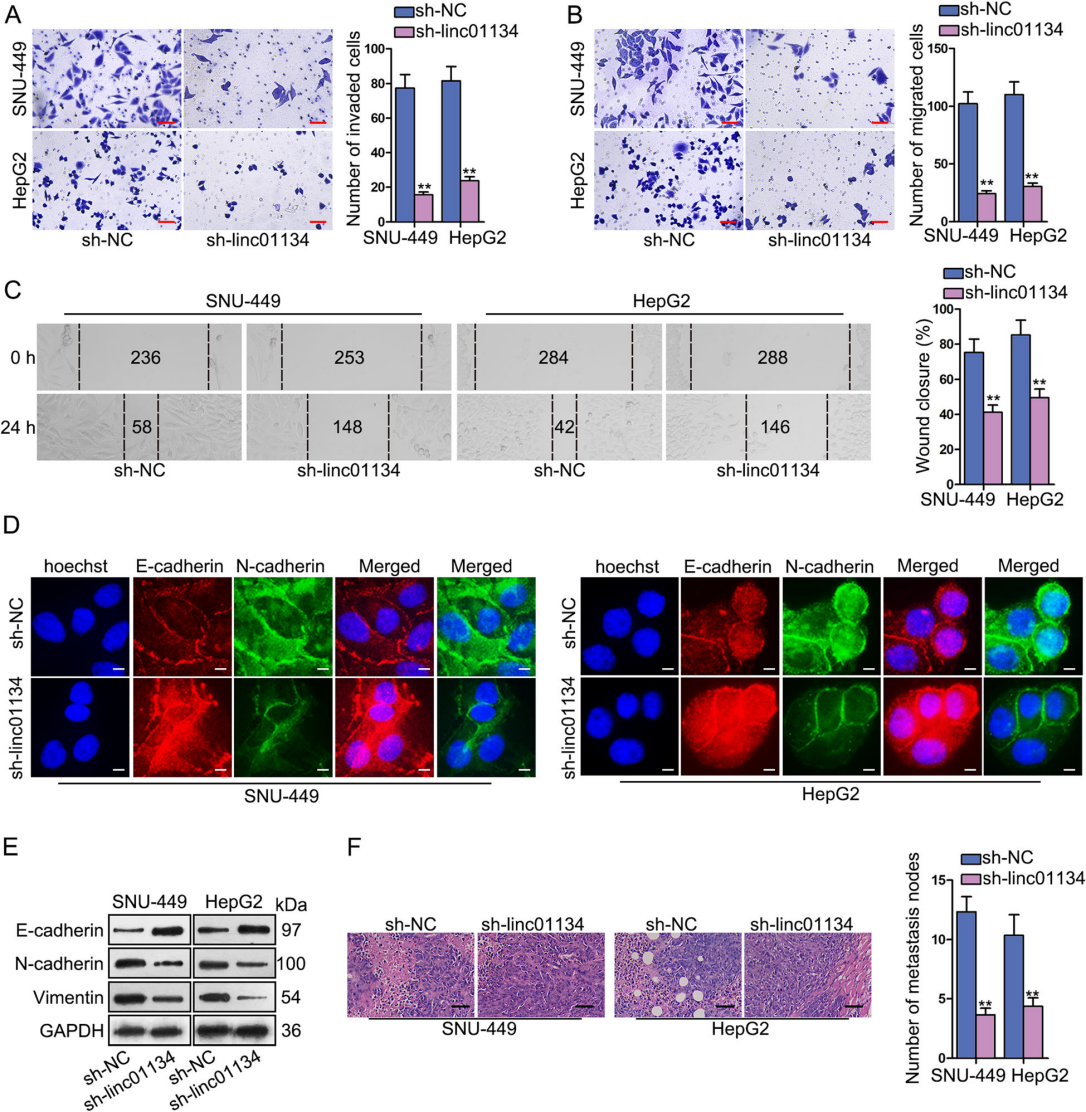
Knockdown of linc01134 can curb HCC cell migration, invasion and EMT process in vitro and impede metastasis in vivo. **a**-**c** Transwell and wound-healing assays were performed to assess cell migration and invasion abilities after knockdown of linc01134. **d** IF analysis was conducted to investigate the expression of E-cadherin and N-cadherin. **e** Western blot was conducted to measure EMT-related protein level. **f** HE staining was conducted to measure the number of metastasis nodes. **P < 0.01

Since migration and metastasis are crucial for HCC deterioration, we evaluated the effects of sh-linc01134 on HCC cell migration and invasion abilities. Transwell assay was conducted to evaluate cell invasion. The result found that knockdown of linc01134 greatly decreased the invaded cells (Fig. 2a). Transwell migration and wound-healing assays demonstrated that silencing linc01134 inhibit cell migration ability to a large extent (Fig. 2b, c).

EMT is a crucial phase that facilitates tumor cell migration and metastasis in HCC. In IF assay, down-regulating linc01134 evidently augmented the expression level of E-cadherin, whereas reduced the level of N-cadherin (Fig. 2d). Based on this phenomenon, we supposed that sh-linc01134 could exert inhibitory effect on EMT process. To further confirm this notion, western blot was performed to measure EMT-related proteins. We identified an increase of E-cadherin, yet a reduction of N-cadherin and Vimentin protein levels (Fig. 2e). Experiments in vivo were conducted to further validate the effects of linc01134 on migration and metastasis. HE staining result demonstrated the decreased metastasis nodes in sh-linc01134 group than control group (Fig. 2f). Altogether, linc01134 knockdown can curb HCC cell migration, invasion and EMT process in vitro and impede metastasis in vivo.

### YY1 stimulates linc01134 transcription

**Fig. 3:**
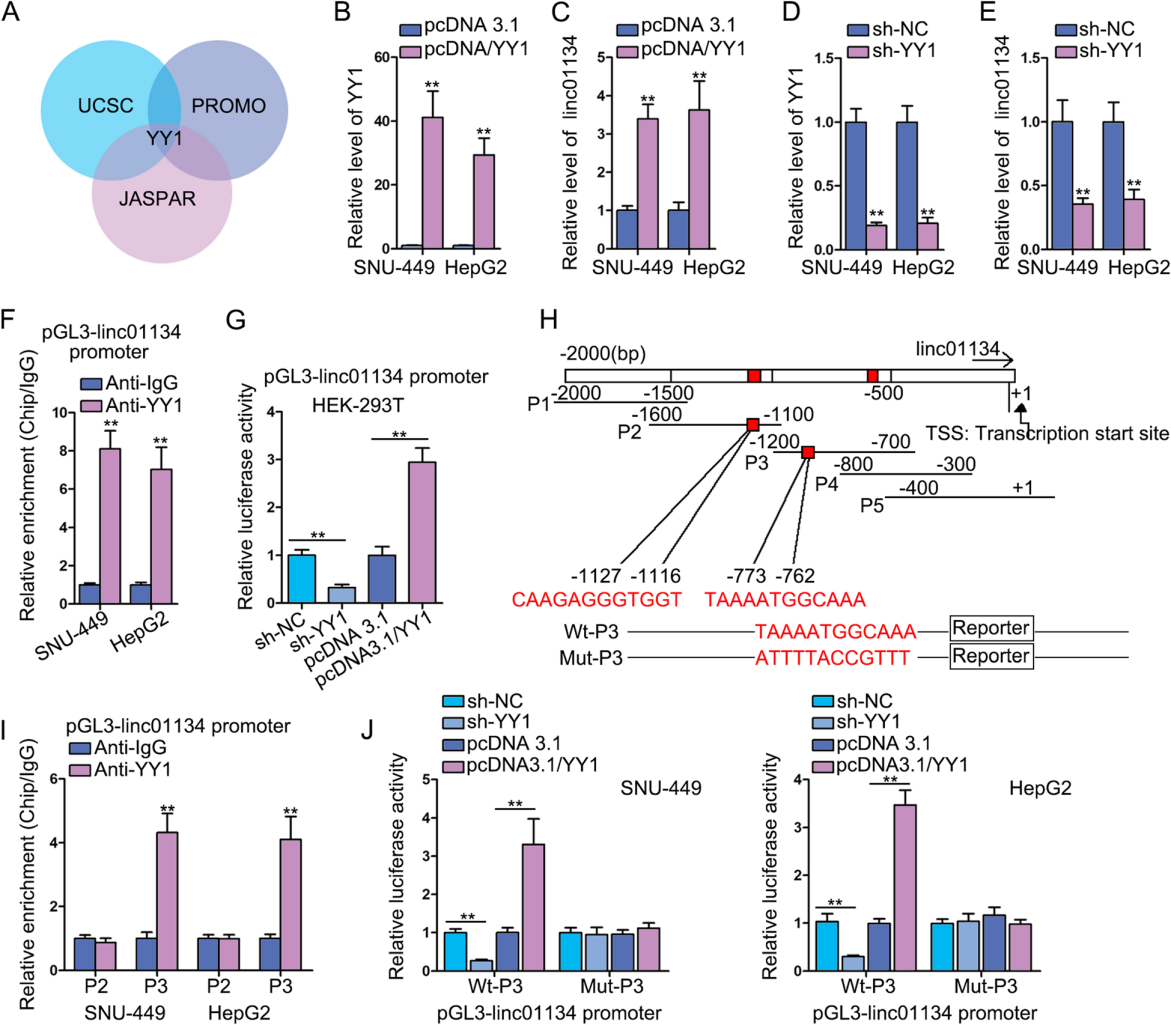
YY1 stimulates linc01134 transcription. **a** Databases predicted YY1 was the transcription factor of linc01134. **b** qRT-PCR analysis was used to determine the overexpression efficiency of YY1. **c** qRT-PCR detected the expression of linc01134 after YY1 overexpression. **d** qRT-PCR was used to determine knockdown efficiency of YY1. **e** qRT-PCR detected the expression of linc01134 after YY1 knockdown. **f** ChIP assay using anti-YY1 and anti-IgG was performed to explore the binding relation between YY1 and linc01134 promoter. **g** luciferase reporter assay detected luciferase activity of linc01134 promoter in transfected HEK-293 T cells. **h** Potential binding sites between YY1 with linc01134 promoter region were predicted. **i** ChIP assay measured the enrichment of linc01134 promoter region in anti-YY1 group. **j** Luciferase reporter assay measured luciferase activity of linc01134 promoter in transfected SNU-449 and HepG2 cells. **P < 0.01

Transcription factor has been found to induce or repress the transcription activity of lncRNA [15]. Given the high expression of linc01134 in HCC, we presumed that transcription factor might play a role. We found potential transcription factor candidate that might interact with linc01134 promoter region using UCSC and Promo database, and noticed that YY1 was the only common transcription factor predicted by UCSC, Promo and JASPAR (Fig. 3a). We then detected the expression of YY1 in HCC tissues and found that YY1 was significantly overexpressed in HCC tissues (Fig. S3A). Correlation analysis revealed a positive relevance between YY1 and linc01134 in HCC tissues (Fig. S3B). To investigate the impacts of YY1 on linc01134 expression, firstly, we overexpressed the expression of YY1 (Fig. 3b). Strikingly, linc01134 expression was increased significantly due to YY1 overexpression (Fig. 3c). Similarly, low YY1 expression was obtained by transfecting sh-YY1 (Fig. 3d) and data showed a significant decline in linc01134 expression after knockdown of YY1 (Fig. 3e). This observation indicated that YY1 indeed regulated linc01134 expression. Secondly, ChIP assay results disclosed that a striking enrichment of linc01134 promoter in anti-YY1 group than anti-IgG control group (Fig. 3f). Moreover, knockdown of YY1 suppressed the promoter luciferase activity, while YY1 overexpression enhanced that of linc01134 promoter, evidenced by luciferase reporter assay (Fig. 3g). This phenomenon indicated that YY1 played a positively regulatory role in linc01134 transcription. Subsequently, we aimed to find out the precise binding site for YY1 at linc01134 promoter region since that two possible binding sites (− 773 to − 762, − 1127 to-1116) were predicted by JASPAR (Fig. 3h). The ChIP data manifested that YY1 could bind to linc01134 P3 promoter region (Fig. 3i). Next, we determined to verify whether − 773 to − 762 sequence existing in P3 that bind to YY1. We constructed Wt-P3 and Mut-P3 for the purpose of luciferase reporter assay. After co-transfecting pcDNA3.1/YY1 with Wt-P3 or Mut-P3, we observed that the promoter activity was greatly enhanced in Wt-P3 group, while no significant change in Mut-P3 group (Fig. 3j). Collectively, we found that YY1 stimulated the transcriptional activity of linc01134 by interacting with linc01134 promoter at − 773 to − 762 site upstream TSS.

### Linc01134 functions as ceRNA to up-regulate IGF2BP1 via sponging miR-324-5p

**Fig. 4:**
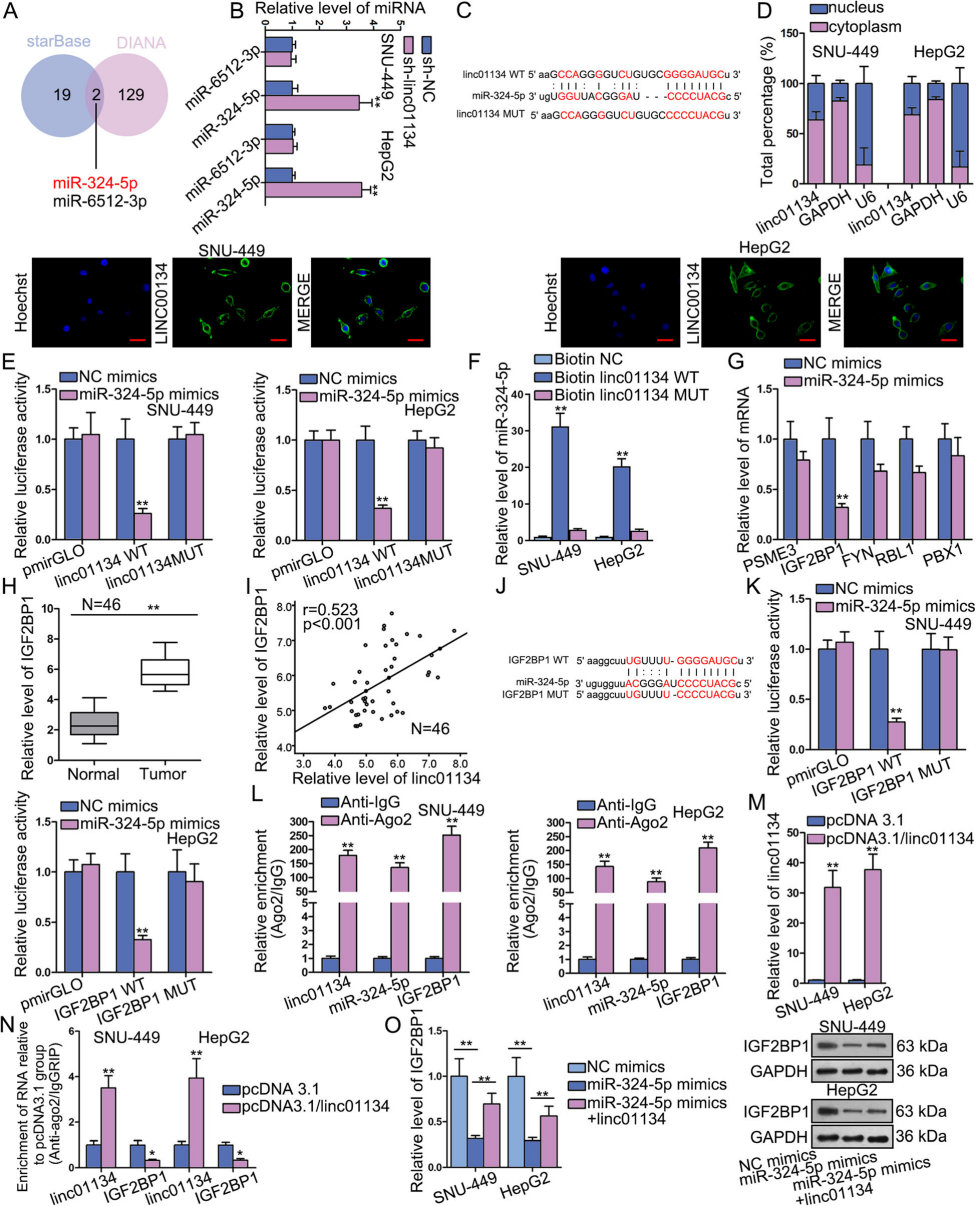
Linc01134 functions as ceRNA to up-regulate IGF2BP1 via sponging miR-324-5p. **a** MiRNAs that can bind to linc01134 were predicted by starBase and DIANA public databases. **b** qRT-PCR was detected the level of miRNAs after silencing linc01134. **c** The binding site between miR-324-5p and linc01134 was predicted by starBase. **d** Subcellular fractionation and FISH assays were conducted to understand the subcellular location of linc01134. **e** Luciferase reporter measured the luciferase activity of linc01134-WT/MUT under miR-324-5p mimics. **f** RNA pull down verified physical interaction between linc01134 and miR-324-5p. **g** qRT-PCR measured the expression of the indicated mRNAs after overexpressing miR-324-5p. **h** qRT-PCR assay was performed to examine the expression of IGF2BP1 in HCC tissues and para-tumor tissues. **i** Pearson correlation was performed to explore the correlation between linc01134 and IGF2BP1. **j** Potential binding site between miR-324-5p and IGF2BP1 was predicted by starBase. **k**-**l** Luciferase reporter and RIP verified the binding relation between IGF2BP1 and miR-324-5p. **m** The transfection efficiency of pcDNA3.1/linc01134 was detected by qRT-PCR. **n** RIP-based PCR evaluated the relative enrichment of linc01134 and IGF2BP1 in anti-Ago2 group. **o** qRT-PCR and western blot were conducted to measure the mRNA and protein levels of IGF2BP1 in transfected cells. *P < 0.05, **P < 0.01

To explore how linc01134 induce cell phenotype changes in HCC, we examined the underlying function mechanism. The ceRNA hypothesis has revealed an important interaction mechanism between RNAs. We testified whether linc01134 functions as a competing endogenous gene in HCC. We searched from starBase (http://starbase.sysu.edu.cn/) and DIANA (http://carolina.imis.athena-innovation.gr/diana_tools/web/index.php?r=site%2Ftools), and detected two shared miRNAs (miR-324-5p and miR-6512-3p) of linc01134 from two databases (Fig. 4a). By knocking down the expression of linc01134, miR-324-5p expression increased sharply but miR-6512-3p expression exhibited no significant change (Fig. 4b). More importantly, a significant down-regulation of miR-324-5p was discovered in HCC tissues, while no distinct difference between the expression of miR-6512-3p in HCC tissues and that of normal one (Fig. S3C). A negative correlation between miR-324-5p and linc01134 in HCC tissues was revealed by Pearson correlation analysis (Fig. S3D), indicating the sponge role of linc01134 possible. Therefore, we selected miR-324-5p as a candidate miRNA to study. We identified a putative binding site of miR-324-5p with linc01134 by browsing starBase (Fig. 4c). Next, subcellular fractionation and FISH assays were conducted to determine the subcellular location of linc01134. The findings showed that linc01134 was principally situated in cytoplasm (Fig. 4d). Luciferase reporter verified the combination between linc01134 and miR-324-5p. It indicated that miR-324-5p mimics significantly dampened the luciferase activity of linc01134-WT, but not linc01134-MUT (Fig. 4e). RNA pull down assay further proved this physical interaction. We observed enriched miR-324-5p was pulled down by biotinylated linc01134-WT compared with linc01134-MUT group (Fig. 4f). Further, we screened the target gene of miR-324-5p from DIANA and found that only five target genes prediction score exceeds 0.8. We transfected miR-324-5p mimics in cells and found that IGF2BP1 expression lessened most dramatically compared with balance mRNA candidates (Fig. 4g). qRT-PCR revealed that IGF2BP1 exhibited significantly high expression in HCC tissues, which is the same status with that of linc01134 (Fig. 4h). In addition, Pearson correlation analysis manifested positive association between IGF2BP1 and linc01134 (Fig. 4i), while a negative one between IGF2BP1 and miR-324-5p (Fig. S3E). Besides, we also discovered that IGF2BP1 expression in HCC tissues was proportional to the level of YY1, the upstream regulator of linc01134, as well (Fig. S3F). The potential binding site of miR-324-5p with IGF2BP1 was found by starBase (Fig. 4j). Luciferase reporter assay manifested that miR-324-5p up-regulation could impair the luciferase activity of IGF2BP1-WT (Fig. 4k). RIP assay indicated the co-existence of linc01134, miR-324-5p and IGF2BP1 in RNA-induced silencing complexes (RISCs) (Fig. 4l). We then examined the overexpression efficiency of linc01134 (Fig. 4m). Interestingly, RIP-based qRT-PCR found that the enrichment of IGF2BP1 in anti-Ago2 group was significantly decreased when linc01134 was overexpressed (Fig. 4n), indicating that linc01134 competitively bound with miR-324-5p against IGF2BP1 in the same RISCs. Furthermore, IGF2BP1 mRNA expression reduced sharply after miR-324-5p was overexpressed, but the falling tendency was reversed by linc01134 up-regulation, which was consistent with changes of IGF2BP1 protein expression by western blot (Fig. 4o). We further found that both IGF2BP1 and linc01134 were enriched in biotinylated miR-324-5p-WT group (Fig. S4A, S4B). Notably, overexpressing linc01134 markedly diminished the abundance of IGF2BP1 in biotinylated miR-324-5p-WT group (Fig. S4C). More importantly, this phenomenon seemed to be resulted by the enhanced enrichment of linc01134 precipitated in anti-Ago2 group when overexpressing linc01134, while Ago2 was the core component of miR-324-5p-guided RISCs (Fig. S4D). Above findings uncovered that cytoplasmic linc01134 functioned as ceRNA to release IGF2BP1 suppressed by miR-324-5p.

### IGF2BP1 enhances the stability of stabilizes of YY1 mRNA in HCC

**Fig. 5:**
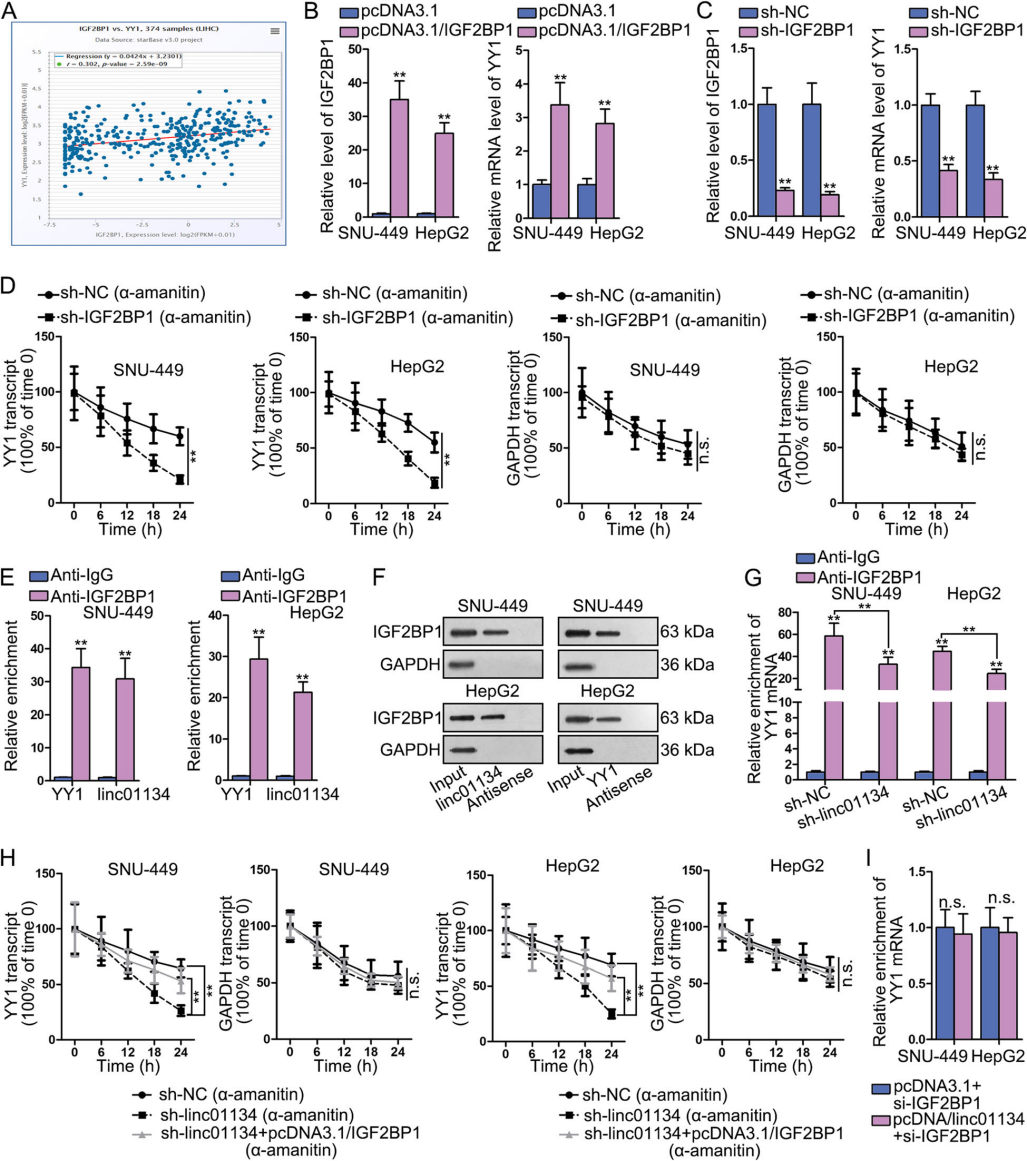
Linc01134 stabilizes YY1 mRNA through recruiting IGF2BP1. **a** Pearson correlation analysis of IGF2BP1 and YY1 in LIHC tissues. **b**-**c** qRT-PCR examined the expression of IGF2BP1 and YY1. **d** qRT-PCR using α-amanitin (50 μM) was performed to measure the effects of knocking down IGF2BP1 on the stability of YY1 and GAPDH (control). **e**-**f**. RIP and RNA pull down were performed to investigate the interaction between IGF2BP1 and YY1 mRNA, as well as between IGF2BP1 and linc01134. **g** RIP assay detected the relative enrichment od YY1 in anti-IGF2BP1 group when silencing linc01134. **h** qRT-PCR using α-amanitin (50 μM) was performed to measure the stability of YY1 mRNA in differently transfected groups. GAPDH was internal reference. **i** qRT-PCR detected the expression of YY1 in differently transfected groups. **P < 0.01. n.s. meant no significance

YY1 has been reported to be overexpressed in multiple cancers, including HCC [16]. Functionally, YY1 could silence tumor-suppressive miRNAs in HCC [11]. Data from starBase found that the expression of IGF2BP1 was positively related to that of YY1 in HCC tissues (Fig. 5a), so was data in 46 HCC tissues obtained clinically. IGF2BP1, a kind of RNA-binding protein (RBP), has been revealed to prevent the degradation of c-myc RNA [17]. To study whether IGF2BP1 affected YY1 expression via preventing its mRNA degradation process, we firstly overexpressed and knocked down IGF2BP1 to observe the change of YY1 mRNA expression. Data displayed that YY1 expression was elevated when IGF2BP1 was up-regulated while was decreased with the down-regulation of IGF2BP1 (Fig. 5b-c). These results verified the positive regulator role of IGF2BP1 on YY1. Thereafter, we examined that whether IGF2BP1 could also modulate YY1 mRNA stability. We knocked down IGF2BP1 in SNU-449 and HepG2 cells, then used qRT-PCR to evaluate the expression changes of YY1 and GAPDH over 24 h period after 48-h α-amanitin (50 μM) treatment. Results demonstrated that knockdown of linc01134 shortened the half-life of YY1 mRNA compared with control group (Fig. 5d). To sum up, IGF2BP1 can enhance the stability of YY1 mRNA and up-regulate its mRNA expression consequently.

### Linc01134 binds to IGF2BP1 protein and strengthen the physical interaction between YY1 mRNA and IGF2BP1 protein

Lately, it has been reported that lncRNAs could affect mRNA stability to regulate gene expression through physical interaction with IGF2BP1 protein [18]. Considering that IGF2BP1 is an RBP, we preliminarily speculated that linc01134 may also bind to IGF2BP1 and impact the association between IGF2BP1 and YY1 mRNA to exert its function in HCC. To verify this hypothesis, we conducted RIP with anti-IGF2BP1 initially. Evident enrichment of linc01134 and YY1 mRNA was observed in anti-IGF2BP1 group compared with anti-IgG group (Fig. 5e). In addition, pull down results revealed that IGF2BP1 protein expression significantly was enriched in linc00134 or YY1 group (sense) compared with negative control group (antisense) (Fig. 5f). Above results manifested the physical interaction between IGF2BP1 and linc01134, as well as between IGF2BP1 and YY1 mRNA.

To investigate whether linc00134 impacted the interaction between IGF2BP1 and YY1 mRNA by binding to IGF2BP1, we silenced linc01134 and conducted RIP assay with anti-IGF2BP1. YY1 mRNA co-precipitated by anti-IGF2BP1 was decreased in linc00134 knockdown group compared with control (Fig. 5g), suggesting that linc01134 enhanced the physical interaction between IGF2BP1 protein and YY1 mRNA.

To further testify whether linc01134 regulated YY1 mRNA stability and mRNA expression via recruiting IGF2BP1, we first performed qRT-PCR after blocking new RNA synthesis with α-amanitin (50 μM) among sh-NC, sh-linc01134 and sh-linc01134 + pcDNA3.1/IGF2BP1 groups. The half-life of YY1 mRNA was significantly shortened by sh-linc01134, but was extended to some extent after co-transfection of pcDNA3.1/IGF2BP1 compared with control group (Fig. 5h). Next, we knocked down IGF2BP1 in pcDNA3.1 or pcDNA3.1/linc01134 transfected cells and found that YY1 mRNA expression was barely changed (Fig. 5i). In a summary, the increase of YY1 mRNA stability regulated by linc01134 was dependent on recruitment of IGF2BP1. Linc01134 interacted with IGF2BP1 protein and strengthen the physical interaction between YY1 mRNA and IGF2BP1 protein.

### YY1 rescues the oncogenic function of linc01134 in HCC

**Fig. 6:**
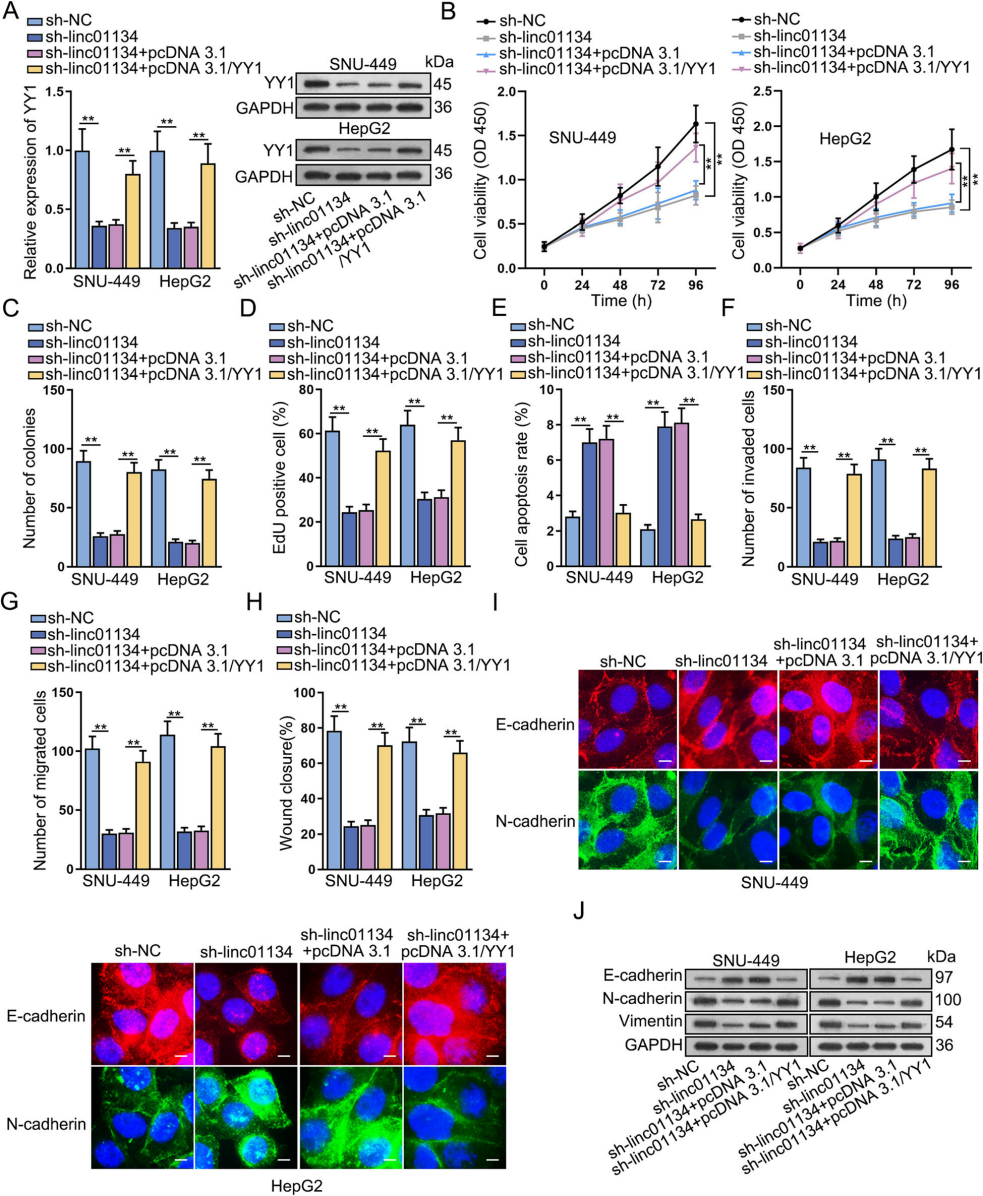
YY1 rescues the oncogenic function of linc01134 in HCC. **a** qRT-PCR tested YY1 mRNA and protein expressions. **b** Cell viability was studied by CCK-8. **c**-**d** Colony formation and EdU were conducted to assess cell proliferation. **e** Flow cytometry was performed to investigate apoptosis. **f** Transwell invasion assay was performed to determine cell invasion ability. **g**-**h** Transwell migration and wound-healing assays were conducted to assess cell migration ability. **i** IF was performed to observe E-cadherin and N-cadherin levels. **j** Western blot assay was conducted to measure EMT-related protein expression. **P < 0.01

A series of rescue experiments were conducted to investigate whether YY1 affect the modulating function of linc01134 in HCC. Results of qRT-PCR and western blot showed that linc01134 knockdown could down-regulate the expression of YY1 mRNA and protein levels, and then overexpression of YY1 reversed this repressing influence (Fig. 6a). CCK-8 result indicated that up-regulation of YY1 could offset the inhibitory effects of sh-linc01134 on cell viability (Fig. 6b). Additionally, colony formation and EdU assays further proved that YY1 enrichment markedly counteracted the obstructive effects of sh-linc01134 on cell proliferation (Fig. 6c, d). Also, cell cycle arrest induced by sh-linc01134 was remedied by overexpressing YY1 (Fig. S5A, S5B). Flow cytometry results manifested that up-regulation of YY1 greatly neutralized the promoting impacts of sh-linc01134 on cell apoptosis (Fig. 6e). On the contrary, YY1 overexpression could greatly countervail the suppressing effects of linc01134 knockdown on cell migration and invasion abilities (Fig. 6f, g, h). YY1 up-regulation restored inhibitory impacts imposed by linc01134 down-regulation on EMT progression, as evidenced by the changes of E-cadherin and N-cadherin levels (Fig. 6i). Besides, western blot results further validated that overexpression of YY1 rescued the impeditive function of linc01134 knockdown on EMT progression (Fig. 6j). These results elucidated that linc01134 affected the proliferative and migrating phenotype of HCC cells via engaging in the miR-324-5p/IGF2BP1/YY1 axis.

**Fig. 7:**
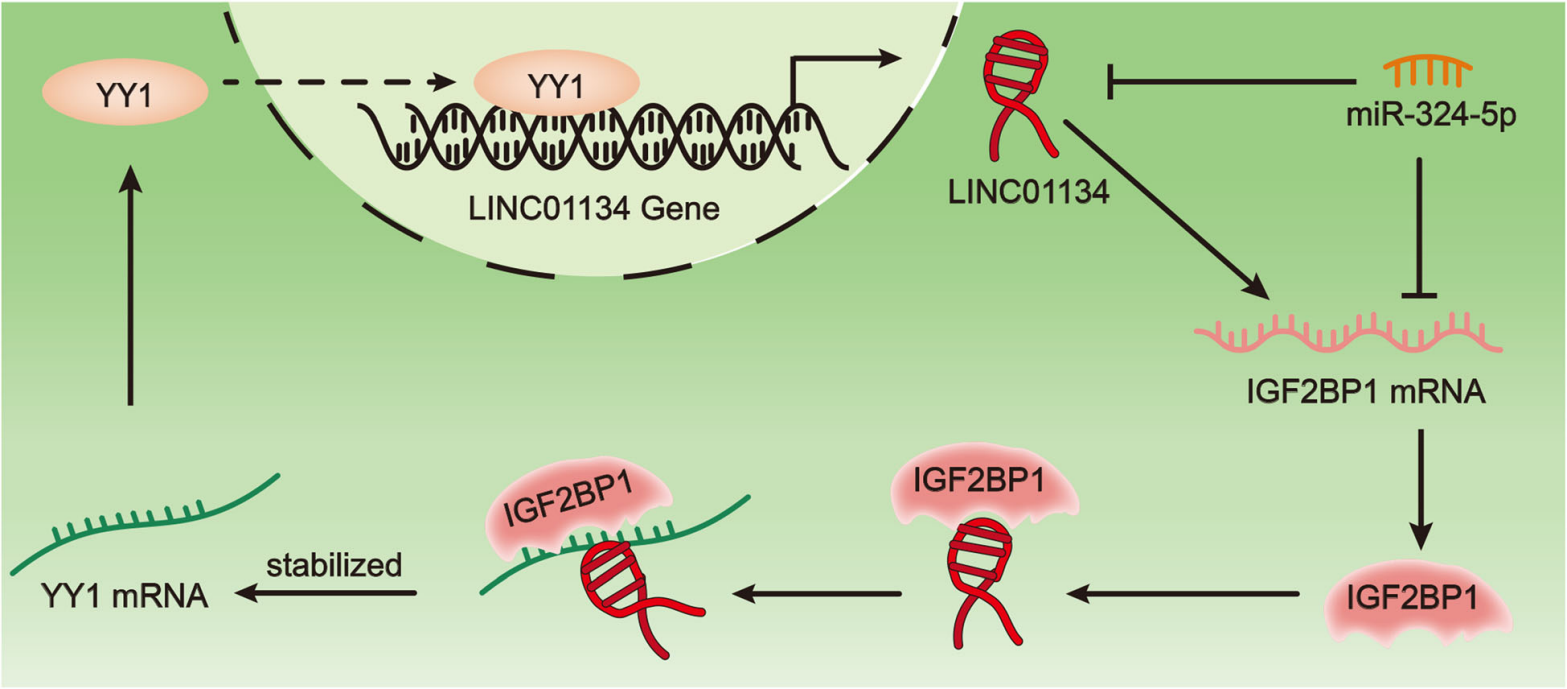
Linc01134/miR-324-5p/IGF2BP1/YY1 forms a positive feedback loop. The figure exhibited the molecular interplay of linc01134/miR-324-5p/IGF2BP1/YY1 in HCC cells

In a summary, YY1 protein can bind to the promoter region of linc01134 and activate the transcription of linc01134 in nucleus. Linc01134 finalizes transcription and transfers into cytoplasm. Cytoplasmic linc01134 competitively binds to miR-324-5p and up-regulates the expression of IGF2BP1. Further, linc01134 associates with IGF2BP1 protein and strengthens the interaction between IGF2BP1 protein and YY1 mRNA, consequently increasing the stability and expression of YY1 mRNA. YY1 mRNA up-regulation induces its protein enrichment. Up-regulated YY1 continuously stimulated linc01134 expression by binding to linc01134 promoter, forming a positive feedback loop in HCC cells (Fig. 7). Linc01134/miR-324-5p/IGF2BP1/YY1 feedback loop promotes cell proliferation, migration and EMT process in HCC. Therefore, targeting linc01134/miR-324-5p/IGF2BP1/YY1 might contribute to providing a new treatment strategy for HCC.

## Discussion

In present study, we examined the expression of the novel long non-coding linc01134 in HCC tissues and para-tumor tissues as well as tumor cells lines and normal cell line. The results manifested that linc01134 was up-regulated in HCC tissues and cell lines, which was attributed to the transcription activity stimulation by YY1. On the one hand, knockdown of linc01134 sharply dampened HCC cell proliferation, yet promoting apoptosis. On the other hand, linc01134 silence deterred cell migration, invasion and EMT progression in vitro. Experiments in vivo further proved that silencing linc01134 could slow down implanted tumor growth and metastasis. Therefore, our findings revealed that linc01134 aggravated the malignant cell phenotype in HCC.

YY1 can function as transcription factor and plays an important regulatory role in various biological processes by binding to DNA and numerous proteins, consequently impacting the pathologic processes of cancers [19–22]. YY1 has been reported to induce lncRNA MCM3AP-AS1 overexpression in lung cancer, and overexpressed MCM3AP-AS1 promoted angiogenesis via targeting KPNA4 through shared miR-340-5p [23]. Moreover, YY1-activated LINC00673 has been revealed to promote proliferative activity of breast cancer cells via regulating MARK4 through sponging miR-515-5p [24]. In current study, we investigated whether YY1 induced linc01134 transcription and affected its molecular regulatory mechanism.

IGF2BP1 belongs to RBPs that can bind to transcripts and regulate RNA stability. Predominantly expressed in the embryo, IGF2BP1 is an essential mediator through the interaction with RNAs in various cancers [25–27]. IGF2BP1 exerts synthesized effects, including cell proliferation, adhesion [28, 29]. In this study, we found that IFG2BP1 was the target of miR-324-5p and its expression was negatively correlated with miR-324-5p expression in HCC tissues. Additionally, linc01134 competitively bound to miR-324-5p against IFG2BP1. The expression of IFG2BP1 mRNA and protein levels were significantly down-regulated after overexpressing miR-324-5p but increased after up-regulating linc01134. Cytoplasmic linc01134 acted as miR-324-5p sponge, consequently elevating the expression of IFG2BP1 mRNA and protein levels. Previous study validated that IGF2BP1 was a crucial protein in modulating EMT progression. IGF2BP1 promoted mesenchymal-like cell properties in tumor-derived cells, and was experimentally proved to serve as a pro-mesenchymal factor [30]. Therefore, it was reasonable to deduce that the linc01134 knockdown repressed EMT progression via diminishing IGF2BP1 expression released from the miR-324-5p induced RISCs. And these evidences suggested that IGF2BP1 was responsible for the acceleration function of linc01134 on EMT progression in HCC.

Apart from the classical ceRNA gene modulation network, increasing lncRNAs have been validated to cooperate with RBPs to modulate mRNA protein stability. For instance, lncRNA THOR was found to up-regulate c-myc expression via strengthening its combination with TGF2BP1 [31]. Interestingly, we found that linc01134 bond to IGF2BP1 and further enhanced physical interaction between IGF2BP1 and YY1 mRNA, finally enhancing the effects of IGF2BP1 on promoting YY1 mRNA stability. Linc01134 depended on IGF2BP1 in stabilizing YY1 mRNA stability. The enhanced YY1 mRNA stability led to the up-regulation of YY1 protein, thus continuously stimulating the transcription of linc00134, forming a positive feedback loop. Besides, the facilitating role of YY1 in the tumorigenesis and EMT of HCC has also been indicated previously [32, 33], which may also explains the contribution of linc01134 to HCC progression. However, due to time limitation, we only explored the YY1 mRNA stability and expression profile changes after the interaction between linc01134 and IGF2BP1. IGF2BP1 can bind to a wide array of other RBPs and forms a distinct ribonucleoprotein complex [30]. Whether linc01134 also impacted other biological functions of IGF2BP1 via physical interaction with IGF2BP1 requires further investigation.

## Conclusions

Taken together, our findings indicate that linc00134 is a meaningful prognostic factor for HCC. Mechanically, YY1-induced linc01134 transcription can promote YY1 mRNA stability and expression via releasing miR-324-5p-targeted IGF2BP1 mRNA and recruiting IGF2BP1, which forms a positive feedback loop in HCC cells. YY1-induced linc01134 up-regulation can promote HCC cell proliferation, migration and invasion, while suppress apoptosis, suggesting that linc01134 might be an essential molecular marker for prognosis and a target for HCC treatment.

## Supplementary information

**Additional file 1: Supplementary Figure 1.** A-C. Relative expression of linc01134 in various human tumor tissues obtained from three databases. D. The non-encoding potential of linc01134 was confirmed by bioinformatics tool.

**Additional file 2: Supplementary Figure 2.** A. PI-FACS analysis was used to detect cell cycle after knockdown of linc01134. B. The mRNA and protein levels of CDK4, cyclin D1 and CDK2 that associated with cell cycle were analyzed. *P < 0.05, **P < 0.01.

**Additional file 3: Supplementary Figure 3.** A. Relative expression of YY1 in HCC and para-tumor tissues was measured by qRT-PCR. B. Pearson correlation analysis between the expression of YY1 and linc01134 in HCC tissues. C. Relative expression of miR-324-5p and miR-6512-3p in HCC and para-tumor tissues was detected by qRT-PCR. D-F. Pearson correlation analysis was used to study the expression correlation between miR-324-5p and linc01134, between IGF2BP1 and miR-324-5p as well as between IGF2BP1 and YY1. **P < 0.01. n.s. meant no significance.

**Additional file 4: Supplementary Figure 4.** A-B. RNA pull down assays measured the enrichment of IGF2BP1/linc01134 in Bio-miR-324-5p-WT/MUT group. C. RNA pull down assays the enrichment of IGF2BP1 in Bio-miR-324-5p-WT/MUT group when overexpressing linc01134. D. RIP assay detected the enrichment of linc01134 in anti-Ago2 group. *P < 0.05, **P < 0.01.

**Additional file 5: Supplementary Figure 5.** A. The cell cycle was detected after co-transfecting pcDNA3.1/YY1 into sh-linc01134 transfected HCC cells. B. The mRNA and protein changes of CDK4, cyclin D1 and CDK2 in differently transfected groups were detected by qRT-PCR and western blot assays. *P < 0.05, **P < 0.01.

## Data Availability

Not applicable.

## Abbreviations

ANOVA: Analysis of variance
ATCC: American type culture collection
CCK-8: Cell counting kit-8
CDK2: Cyclin-dependent kinase-4
CDK4: Cyclin-dependent kinase-4
ceRNA: Competing endogenous RNA
ChIP: Chromatin immunoprecipitation
DAPI: 4′,6-diamidino-2-phenylindole
DMEM: Dulbecco’s modified eagle medium
EdU: 5-ethynyl-2′-deoxyuridine
EMT: Epithelial-mesenchymal transition
EZH2: Enhancer of zeste homolog 2
FBS: Fetal bovine serum
FISH: Fluorescence in situ hybridization
GLI1: Glioma-associated oncogene homolog 1
H3K27me3: Histone3 lysine27 tri-methylation
HCC: Hepatocellular carcinoma
HE Staining: Haematoxylin and eosin
IF: Immunofluorescence
IGF2BP1: Insulin-like growth factor 2 mRNA binding protein 1
IHC: Immunohistochemistry
KPNA4: Karyopherin α4
LIHC: Liver hepatocellular carcinoma
lncRNAs: Long non-coding RNAs
MARK4: Microtubule affinity regulating kinase 4
MCM3AP-AS1: Minichromosome maintenance complex component 3 associated protein antisense RNA 1
microRNAs: MiRNAs
mRNA: Messenger RNA
NF-κB: Nuclear factor kappa B
PBS: Phosphate-buffered saline
PVDF: Polyvinylidene fluoride
qRT-PCR: Quantitative real-time polymerase chain reaction
RBPs: RNA binding proteins
RIP: RNA immunoprecipitation
SDS PAGE: Sodium dodecyl sulfate polyacrylamide gel electrophoresis
TSS: Transcription termination site
VEGFA: Vascular endothelial growth factor
YY1: Yin Yang-1
ZFAS1: Zinc finger antisense 1
