# Supplementary figures and images for: Molecular interplay between linc01134 and YY1 dictates hepatocellular carcinoma progression
Source: J Exp Clin Cancer Res. 2020 Apr 9;39:61. doi: 10.1186/s13046-020-01551-9 (PMC7146959; doi:10.1186/s13046-020-01551-9)

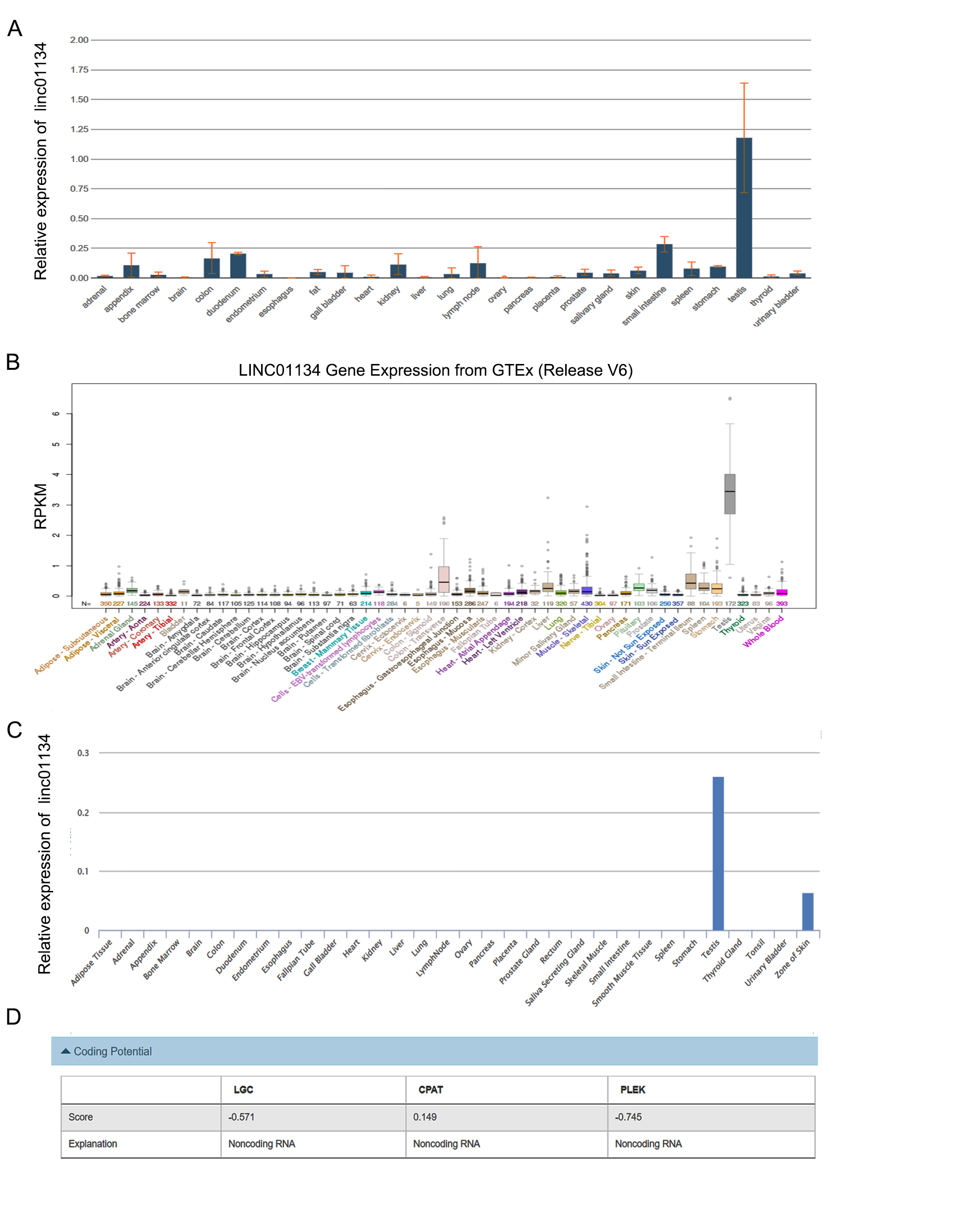

Supplement: Supplementary file 1 — Additional file 1: Supplementary Figure 1. A-C. Relative expression of linc01134 in various human tumor tissues obtained from three databases. D. The non-encoding potential of linc01134 was confirmed by bioinformatics tool. [file 13046_2020_1551_MOESM1_ESM.tif]

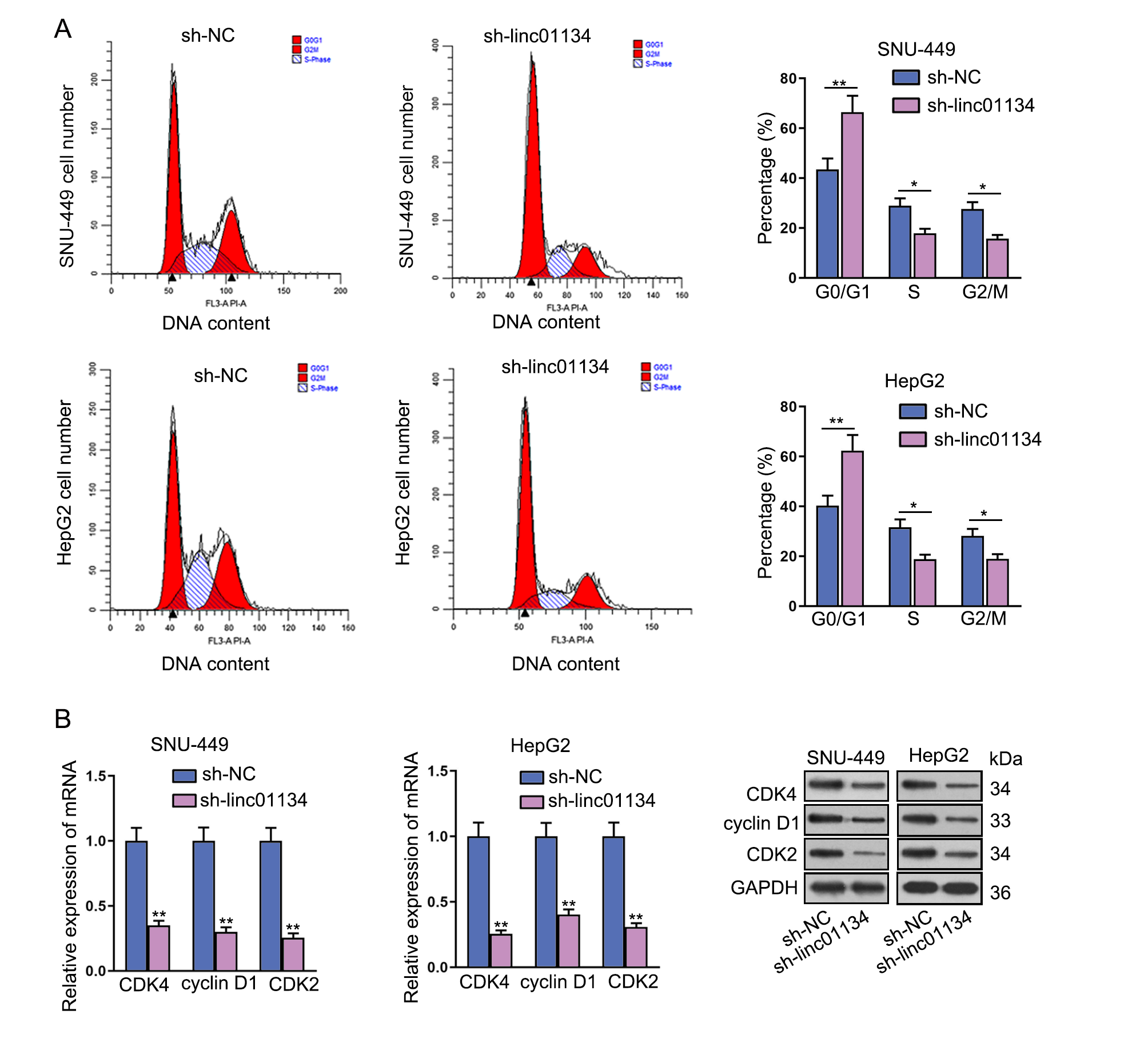

Supplement: Supplementary file 2 — Additional file 2: Supplementary Figure 2. A. PI-FACS analysis was used to detect cell cycle after knockdown of linc01134. B. The mRNA and protein levels of CDK4, cyclin D1 and CDK2 that associated with cell cycle were analyzed. *P < 0.05, **P < 0.01. [file 13046_2020_1551_MOESM2_ESM.tif]

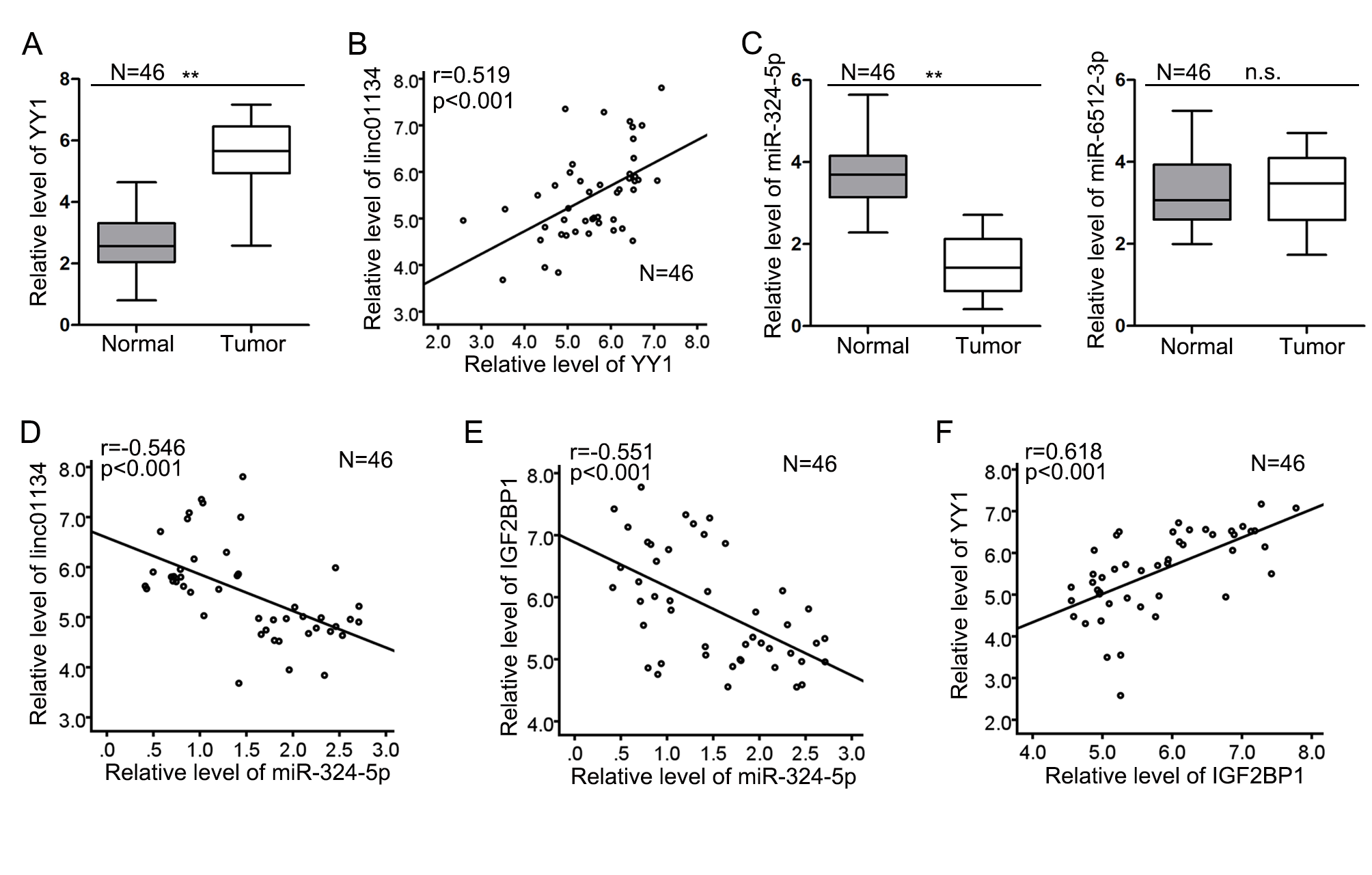

Supplement: Supplementary file 3 — Additional file 3: Supplementary Figure 3. A. Relative expression of YY1 in HCC and para-tumor tissues was measured by qRT-PCR. B. Pearson correlation analysis between the expression of YY1 and linc01134 in HCC tissues. C. Relative expression of miR-324-5p and miR-6512-3p in HCC and para-tumor tissues was detected by qRT-PCR. D-F. Pearson correlation analysis was used to study the expression correlation between miR-324-5p and linc01134, between IGF2BP1 and miR-324-5p as well as between IGF2BP1 and YY1. **P < 0.01. n.s. meant no significance. [file 13046_2020_1551_MOESM3_ESM.tif]

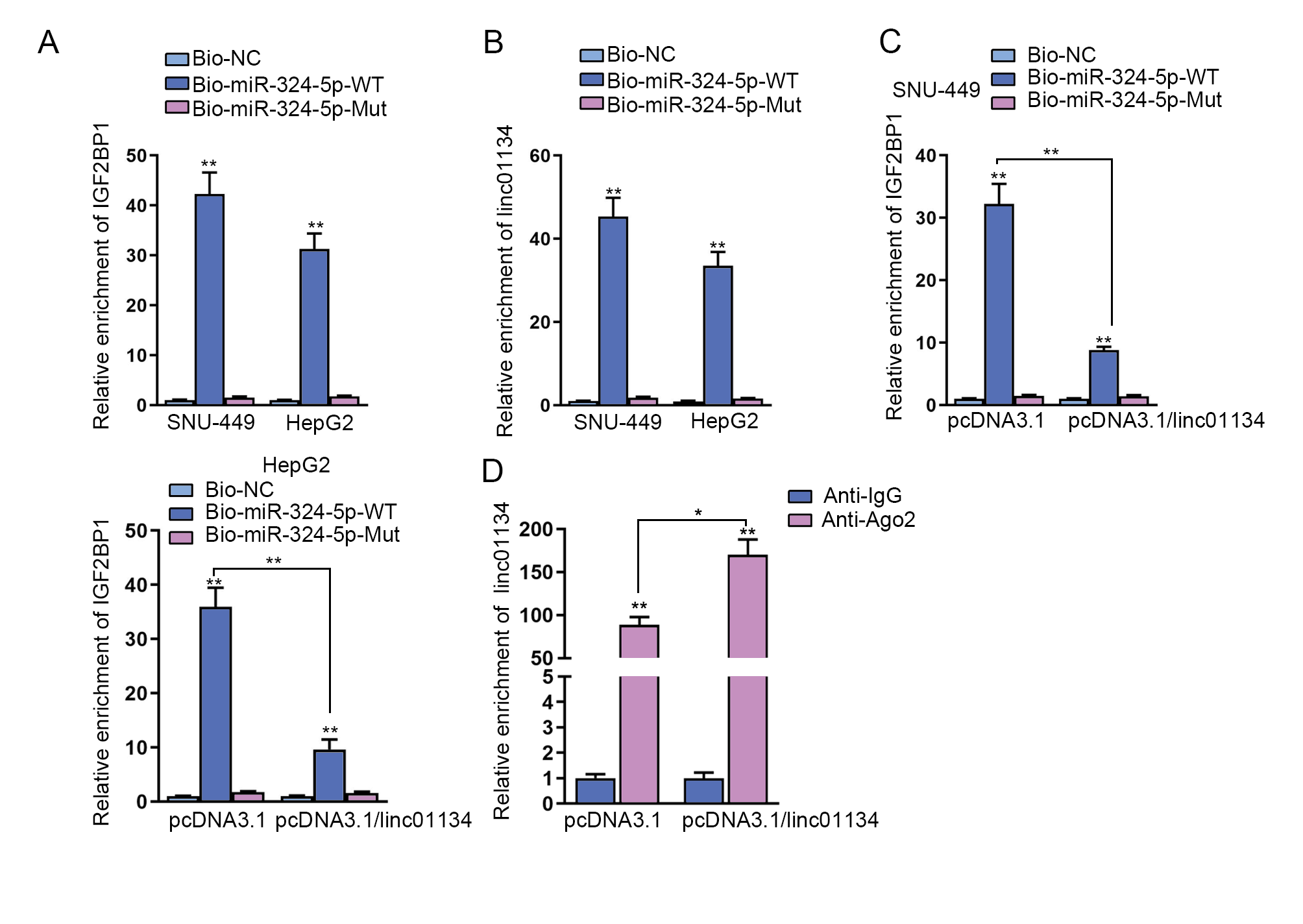

Supplement: Supplementary file 4 — Additional file 4: Supplementary Figure 4. A-B. RNA pull down assays measured the enrichment of IGF2BP1/linc01134 in Bio-miR-324-5p-WT/MUT group. C. RNA pull down assays the enrichment of IGF2BP1 in Bio-miR-324-5p-WT/MUT group when overexpressing linc01134. D. RIP assay detected the enrichment of linc01134 in anti-Ago2 group. *P < 0.05, **P < 0.01. [file 13046_2020_1551_MOESM4_ESM.tif]

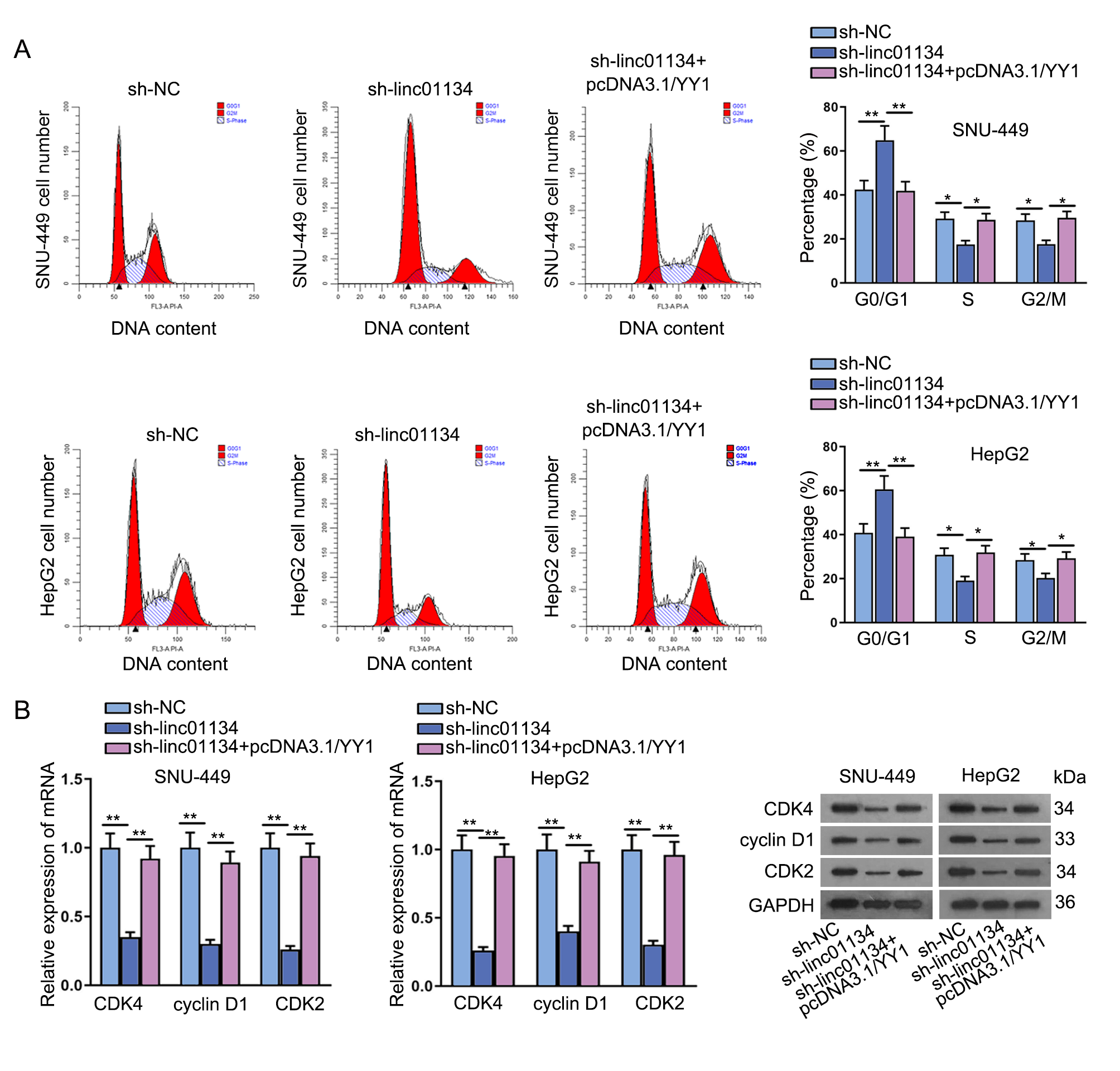

Supplement: Supplementary file 5 — Additional file 5: Supplementary Figure 5. A. The cell cycle was detected after co-transfecting pcDNA3.1/YY1 into sh-linc01134 transfected HCC cells. B. The mRNA and protein changes of CDK4, cyclin D1 and CDK2 in differently transfected groups were detected by qRT-PCR and western blot assays. *P < 0.05, **P < 0.01. [file 13046_2020_1551_MOESM5_ESM.tif]
